# Who Will Voice the Nurse?

**Published:** 1921-01-22

**Authors:** 


### LORD CAVE'S COMMISSION.

### Who Will Voice the Nurse?

APROPOS of the fact that Lord Cave has been appointed Chairman to a Committee whose function it is to inquire into the financial position of voluntary hospitals, it is devoutly to be hoped that in the Report the ultimate fate of these institutions is not already determined. The Chairman is a Lord of Appeal, and has a wide experience of affairs, having in turn been Judge, Member of Parliament, Solicitor-General, and Home Secretary. His Committee will be appointed by the Minister of Health, and the terms of reference do not appear to have been yet finally decided. It cannot be too strongly urged that this inquiry should be broad and far-reaching. On this subject there is a great deal to be said. The war has changed the face of the world, and there are a great many people of the pre-war epoch who fail to apprehend the fact. It is an indisputable fact that since the war ended there has been no propaganda worth the name to match the new conditions. Under the old regime propaganda became stereotyped. One class only of the community contributed to an appreciable extent, and they were easily reached. A personal letter, or an appeal through *The Times*, went a long way.

### THE CAUSE OF CONTRIBUTIONS.

Contributions flowed in owing to two causes. First, those who gave possessed the means to enable them to do so, and in the majority of instances the giving tradition had been handed down to them with their wealth. The other reason that prompted giving was the fact that the voluntary hospital ministered to the wants of the very poor. But circumstances have altered. The people who were well off before the war have in a great many cases become poor, and a new set, differently bred, have acquired riches. They have never been trained to give, and they do not understand public benefaction. Then the sick who seek hospital relief are very often workers in receipt of high wages, who should neither expect nor receive free treatment in their homes or in the hospital. The public understand this in a general way, but only in a general way, and what I maintain is that the facts have not been analysed, tabulated, and published. Even very well-informed people have only a vague impression of what the changes that have taken place involve. And no appeal has been made to those who have never contributed but who can afford to do so. My conviction is that those who are looking about for municipal support have too quickly abandoned the quest for funds. They find, naturally, that the old methods fail, and they lack the imagination to devise new means suited to new circumstances.

For example. The best way to begin to train the new rich to give is to get at their children. Children love to pay a visit to a hospital, and visiting a hospital does them a world of good. It awakens their sympathies, and reminds them that there are those whose lives need brightening. Hospitals ought to open certain of their wards to the public, and see that the schools take advantage of the opportunity of visiting them. Handing a collecting-card to a child who has never visited a hospital is the way to failure. I feel sure that through the children hospitals might be able to ensure the endowment of a large number of beds, and, what is of greater prospective value, the public would thus become educated as to the value of the voluntary hospital to the nation.

### A PROPAGANDA BUREAU?

There is, as I have observed, a great deal to be said about propaganda, and I merely cite this as an example. If it is ever undertaken as the necessity demands it should be done in a businesslike fashion. Hospitals should establish a propaganda bureau, and experts should be employed. The method now in vogue is as feeble as it is futile, and until other methods are tried and fail it would be folly to throw the voluntary system overboard.

I am, however, more particularly concerned with another matter. What about the nurse? I am addressing this interrogation to the nurses' leaders. The voluntary hospital consists of three essential factors—the building, the doctor, and the nurse. There is, of course, the equipment, but I throw this in with the building. The question at issue is, Will the case of the nurse be efficiently and effectually presented to Lord Cave; and, if it is, who is going to undertake the duty? The case of the nurse is that she has been, up to the present hour, a vital part of the voluntary contributions by which the hospitals are maintained. Her's is noble work; it is national work; and British working men and women do not want to employ underpaid and overwrought women to nurse their sick back to health.

It appears to me that Lord Cave's Committee must take account of this question. It is imperative. But who is to present the evidence? Who, in the first instance, is to prepare it? The preparing and presenting of the case require champions of no mean ability.

IERNE.

